# Post-Embolization Syndrome Complicated by Hypertensive Emergency and Severely Elevated Transaminases

**DOI:** 10.7759/cureus.15446

**Published:** 2021-06-04

**Authors:** Simon Kashfi, Elizabeth Murdakhayev, Razia Rehmani, Shorabh Sharma

**Affiliations:** 1 Internal Medicine, City University of New York (CUNY) School of Medicine, New York, USA; 2 Radiology, St. Barnabas Hospital Health System, Bronx, USA; 3 Internal Medicine, St. Barnabas Hospital Health System, Bronx, USA; 4 Internal Medicine, Hospitalist, Bronx, USA

**Keywords:** post embolization syndrome, hepatocellular carcinoma (hcc), hypertensive emergency, transarterial chemoembolization (tace), transaminase

## Abstract

Transarterial chemoembolization (TACE) is a procedure reserved for the treatment of hepatocellular cancer that is unresectable through surgery. It combines both embolization and chemotherapy by injecting chemotherapy via a catheter directed at the tumor and then blocking the artery to prevent blood flow to the tumor. We present the case of a 69-year-old man who experienced post-embolization syndrome (PES) with a hypertensive emergency and elevated liver transaminases following his TACE procedure. Imaging combined with clinical assessment was necessary to determine whether the patient was experiencing a ruptured hepatic abscess or PES, as both are potential complications of TACE. The patient was ultimately managed with supportive care and discharged after several days.

## Introduction

The incidence of hepatocellular carcinoma (HCC) depends on the geographic location and population. The incidence in the US has increased to 6.2/100,000 in 2011, with African-Americans and Hispanics aged 45-65 years affected the most [1,2]. The estimated incidence of HCC in 2020 was 32,108 cases [3]. Common risk factors for HCC in the US are hepatitis B, hepatitis C, and alcoholic and non-alcoholic fatty liver disease [1]. Treatment for HCC largely depends on the patient’s functional status and tumor burden. The Barcelona Clinic Liver Cancer (BCLC) staging system offers an algorithm that guides treatment strategies in patients with HCC [4]. For patients with earlier stage HCC, defined by a single tumor ≤ 2cm or HCC in situ, hepatic resection or transplant is preferred. However, most patients present with later stages of HCC, which are often unresectable. For intermediate stage HCC patients, defined as asymptomatic, large, or multifocal HCCs without evidence of vascular invasion or extrahepatic metastasis, transarterial chemoembolization (TACE) is the preferred treatment [4,5]. The TACE procedure involves embolization of the arterial supply to the tumor combined with highly concentrated local chemotherapeutic drugs. The most common side effect of TACE, occurring in 60-80% of patients, is post-embolization syndrome (PES) [6]. This is usually characterized by abdominal pain, nausea, fever, and elevated transaminases that occur 24-72 hours after the procedure [6,7]. However, more serious complications such as acute liver decompensation (encephalopathy, ascites) can occur [5,8,9]. PES has been defined as a clinical diagnosis by some, and thus they do not report specific liver function enzymes [5,8]. However, elevation of liver transaminases occurs in a majority of cases, and there are no universal lab criteria to define PES [7]. Some studies have defined PES as an increase of aspartate aminotransferase (AST) to above 100 IU/L with at least doubling of its baseline value [10,11]. We present a case of TACE with PES complicated by hypertensive emergency and severely elevated transaminases.

## Case presentation

A 69-year-old African American male with a history of hypertension, HIV, and treated hepatitis C in 2015 that was not followed with serial ultrasounds presented to the emergency department with sudden, continuous, cramping right upper quadrant abdominal pain that radiated to the back and was worsened with deep inspiration. The pain started while playing with his granddaughter. Associated symptoms included a five-pound weight loss in two months and unremitting bitemporal headache. He presented with a systolic blood pressure (SBP) of >200 mmHg and diastolic blood pressure of >110 mmHg.

**Figure 1:**
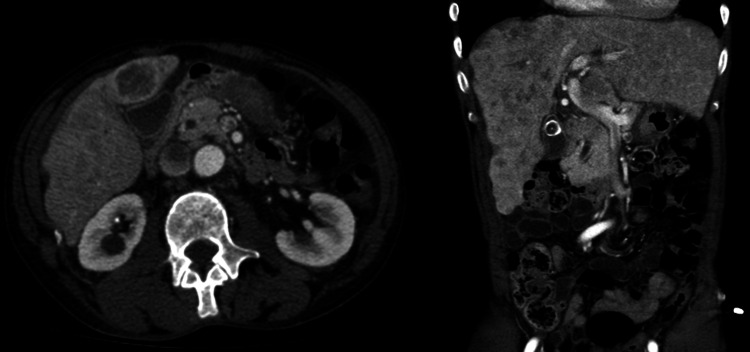
Axial and coronal images from a contrast-enhanced CT scan of the abdomen. Multiple hypoattenuating foci throughout the liver compatible with multiple hepatomas before TACE can be seen. TACE, transarterial chemoembolization

Right upper quadrant ultrasound revealed hepatomegaly with multiple masses suspicious for metastasis, a thickened gallbladder, calcified gallstones, and retroperitoneal lymphadenopathy. The left lobar hepatic mass measured 2.4 x 2.1 x 3.6 cm and right lobar masses measured 4.5 x 2.9 cm, 3.6 x 2.7 cm, 2.4 x 2.2 cm, and 3 x 2.3 cm. On triple-phase CT of the liver (Figure 1), findings were consistent with the ultrasound, and lucent lesions were also found in both iliac bones. Labs at this time were significant for an alpha fetoprotein (AFP) of 3,498 ng/mL and a carcinoembryonic antigen (CEA) of 3.1 ng/mL. The patient was diagnosed with HCC, and TACE procedure was scheduled in three weeks.

**Figure 2:**
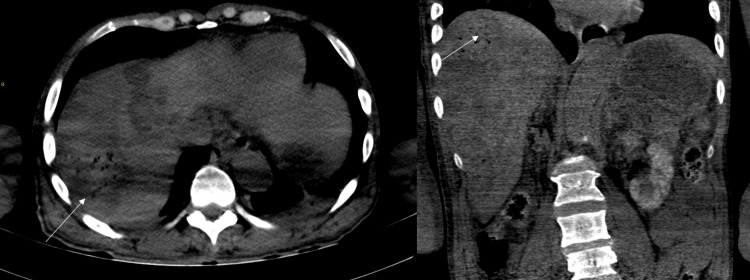
Noncontrast axial and coronal CT scan images. Numerous hypoattenuating lesions throughout the liver with new foci of gas (arrow) can be seen in the right hepatic lobe after TACE in the presence of leukocytosis, and pain is suggestive of post-embolization syndrome. TACE, transarterial chemoembolization

The patient underwent outpatient TACE procedure with 75 mg doxorubicin with LC beads to the right hepatic artery. Immediately post-procedure, the patient was persistently hypertensive after labetalol and verapamil IV push with SBP in 220s mmHg along with persistent nausea and was sent to the ED. In the ED, the patient had continued right upper quadrant and epigastric pain as well as hypertension (blood pressure: 250s/130s mmHg). EKG showed normal sinus rhythm with a prolonged QTc of 495 ms with signs of left ventricular hypertrophy. Troponin was negative, and chest X-ray showed no evidence of infiltrates or pleural effusion. CT angiogram of the abdomen showed hypoattenuated territories consistent with TACE, cholelithiasis, right nephrolithiasis, prominent para-celiac lymph node, and distended bladder. The imaging (Figure 2) was concerning for possible hepatic abscesses, and thus clinical correlation was required to determine ruptured hepatic abscess versus PES. The patient was admitted to the hospital and was treated symptomatically. His main complaint was severe right upper quadrant pain, which gradually improved until discharge after five days. Table 1 shows relevant lab values that were tracked during the patient's hospital stay.

**Table 1:** Laboratory values before and during admission POD, postoperative day; WBC, white blood cells; AST, aspartate aminotransferase; ALT, alanine aminotransferase; ALP, alkaline phosphatase

	Baseline	POD 0	POD 1	POD 2	POD 3	POD 4	Three weeks after discharge
WBC (10^3^/uL)	7.9	9.3	12.2	20.4	21.7	18.8	8.6
AST (IU/L)	32	66	263	700	281	132	52
ALT (IU/L)	14	25	100	311	231	149	40
ALP (IU/L)	106	173	179	253	308	267	181
Bilirubin (mg/dL)	0.4	1.3	0.5	0.6	0.9	0.7	0.5

## Discussion

Our patient was in BCLC stage B, which is classified by multiple hepatic tumors without evidence of extrahepatic spread [4]. There are some studies that calculate the Child-Pugh score, which calculates hepatic function and estimates severity of cirrhosis [12]. These studies used the score as inclusion criteria for TACE, using only patients in Child-Pugh class A or B [13,14]. Patients in class A may have better long-term outcomes than those in class B [13,15]. Our patient’s Child-Pugh score was 7, putting him in class B.

There are generally two variations to the TACE procedure, which are the conventional and drug-eluting bead (DEB) TACE. The conventional TACE procedure involves intra-arterial chemotherapy - usually doxorubicin or cisplatin - followed by embolization with gel-foam particles, while the DEB-TACE procedure incorporates chemotherapy-eluting beads. The DEB-TACE procedure is thought to enhance the anti-tumor effect because of the slow, controlled release of the drug [9]. The DEB-TACE procedure has also been shown to have fewer and milder side effects than the conventional TACE procedure [13,16]. The more serious complications such as acute liver decompensation (encephalopathy, ascites) occur in 0.1-3% of DEB-TACE procedures. Biliary complications occur in 2-10% of patients and GI complications in 1-5% [9]. Liver abscess can also occur up to 10% of cases, making clinical correlation of diagnostic imaging necessary [9].

Our patient is unique in the sense that his hypertension started immediately post-operatively. Additionally, his transaminase levels were uniquely high on post-operative day 2. Comparison of our patient’s liver function enzymes is difficult due to the variability in defining PES, and not all studies include liver function enzymes. Comparison to one case [7] reveals that our patient had very high transaminases and white blood cell count with lower alkaline phosphatase (ALP) and bilirubin. Additionally, one study from 256 TACE procedures shows that the median increase in AST was 158 IU/L, in ALP was 15 IU/L, and in bilirubin was 11 mg/dL [10]. The increase in AST in our patient was 668 IU/L, and the increase in ALP was 147 IU/L. These increases are appreciably larger than what was found in that study, though the increase in our patient’s bilirubin was lower than the median in the study. It should be noted that it is unclear when in the post-operative course the data were collected, making it difficult to assess whether this was the maximum increase in liver function enzymes. Since PES is a self-limiting syndrome, our patient was treated symptomatically and was discharged on post-operative day 4. He was admitted again to the hospital three weeks after discharge for an unrelated episode of hyperosmolar hyperglycemic syndrome, and his liver function enzymes had nearly returned to pre-operative levels.

## Conclusions

PES is a common complication of TACE usually characterized by abdominal pain, nausea, fever, and elevated transaminases one to three days after the procedure. The case presented here demonstrates that PES can occur immediately after the procedure and may be complicated by severe hypertensive emergency, elevated transaminases, and afebrile leukocytosis, but it is self-limiting. Clinicians should be aware of such a presentation and provide supportive care.
